# Supplementary material for: Transport mechanism of P4 ATPase phosphatidylcholine flippases
Source: eLife. 2020 Dec 15;9:e62163. doi: 10.7554/eLife.62163 (PMC7773333; doi:10.7554/eLife.62163)
Supplement: Supplementary file 1. [file elife-62163-supp1.docx]

**Supplemental Table 1. Cryo-EM data collection, refinement, and validations.**

|  | **Dnf1–Lem3** | | | **Dnf2–Lem3** | | | | |
| --- | --- | --- | --- | --- | --- | --- | --- | --- |
|  | **Apo** | **E1-ADP** | **E2P** | **Apo** | **E1-ATP** | **E1-ADP** | **E2P- transition** | **E2P** |
| **Data collection and processing** |  |  |  |  |  |  |  |  |
| Magnification (k) | 130 | 130 | 130 | 130 | 130 | 130 | 130 | 130 |
| Voltage (kV) | 300 | 300 | 300 | 300 | 300 | 300 | 300 | 300 |
| Electron expo- sure (e^–^/Å^2^) | 64 | 64 | 64 | 60 | 60 | 60 | 60 | 60 |
| Defocus range  (-μm) | 1.0-2.0 | 1.0-2.0 | 1.0-2.0 | 1.0-2.0 | 1.5-3.0 | 1.5-3.0 | 1.5-3.0 | 1.5-3.0 |
| Pixel size (Å) | 0.826 | 0.826 | 0.826 | 1.029 | 0.826 | 0.826 | 0.826 | 0.826 |
| Symmetry imposed | C1 | C1 | C1 | C1 | C1 | C1 | C1 | C1 |
| Micrograph # | 4,074 | 6,038 | 4,764 | 1,438 | 3,665 | 5683 | 5683 | 3260 |
| Initial particle # | 1,358,323 | 2,257,199 | 2,094,437 | 509,555 | 844,464 | 1,010,708 | 1,010,708 | 1,095,601 |
| Final particle # | 473,761 | 716,510 | 590,043 | 190,753 | 358,687 | 266,218 | 127,442 | 763,661 |
| Map resolution (Å) | 3.00 | 3.25 | 2.82 | 3.08 | 3.85 | 4.05 | 3.98 | 3.50 |
| FSC threshold | 0.143 | 0.143 | 0.143 | 0.143 | 0.143 | 0.143 | 0.143 | 0.143 |
| Map resolution (Å) | 3.12 | 3.25 | 2.82 | 3.08 | 3.85 | 4.05 | 3.98 | 3.50 |
|  |  |  |  |  |  |  |  |  |
| **Refinement** |  |  |  |  |  |  |  |  |
| Map sharpening *B* factor (Å^2^) | 116.5 | 178.9 | 116.2 | 102.5 | 195.8 | 243.3 | 197.8 | 180.0 |
| Model compo- sition  Non-hydrogen atoms  Protein residues  Ligands & glycans | 11150  1332  10 | 11235  1322  14 | 13121  1552  14 | 10369  1247  17 | 10684  1274  20 | 11960  1471  14 | 12521  1534  15 | 12879  1545  18 |
| *B* factors (Å^2^)  Protein  Ligand | 81  67 | 39  45 | 47  34 | 86  56 | 48  47 | 175  174 | 166  158 | 58  50 |
| R.m.s. deviations  Bond lengths (Å)  Bond angles (°) | 0.01  0.8 | 0.01  0.9 | 0.01  1.1 | 0.004  0.6 | 0.003  0.6 | 0.002  0.6 | 0.002  0.6 | 0.003  0.6 |
| Validation  MolProbity score  Clashscore  Poor rotamers (%) | 2.4  13  1 | 2.5  11  1 | 2.0  11  1 | 2.1  14  1 | 2.1  12  0 | 2.1  12  0 | 2.0  13  0 | 2.1  13  1 |
| Ramachandran plot  Favored (%)  Allowed (%)  Disallowed (%) | 93.0  7.0  0 | 91.9  8.1  0 | 95.6  4.4  0 | 93.0  6.9  0 | 90.7  9.2  0 | 93.1  6.9  0 | 93.9  5.9  0 | 92.9  7.1  0 |
