# Supplementary material for: Transport mechanism of P4 ATPase phosphatidylcholine flippases
Source: eLife. 2020 Dec 15;9:e62163. doi: 10.7554/eLife.62163 (PMC7773333; doi:10.7554/eLife.62163)
Supplement: Supplementary file 2. [file elife-62163-supp2.docx]

**Supplementary File 2. List of plasmids used in the study.**

| **Plasmid** | **Source** |
| --- | --- |
| pRS313 | (Sikorski and Hieter, 1989) |
| pRS425 | (Sikorski and Hieter, 1989) |
| pRS313-Dnf1 | (Baldridge and Graham, 2012) |
| pRS313-Dnf1 R264A |  |
| pRS313-Dnf1 Y633A |  |
| pRS313-Dnf1 Y633G |  |
| pRS313-Dnf1 T648A |  |
| pRS313-Dnf1 T648V |  |
| pRS313-Dnf1 W652A |  |
| pRS313-Dnf1 W652S |  |
| pRS313-Dnf1 Q610A |  |
| pRS313-Dnf1 S611A |  |
| pRS313-Dnf1 YQS-FSN | (Baldridge and Graham, 2012) |
| pRS313-Dnf1 N1226A |  |
| pRS313-Dnf1 (692-737)∆ |  |
| pRS416-GFP-Dnf1 | (Baldridge and Graham, 2012) |
| pRS416-GFP-Dnf1 R264A |  |
| pRS416-GFP-Dnf1 W652A |  |
| pRS416-GFP-Dnf1 W652S |  |
| pRS416-GFP-Dnf1 Q610A |  |
| pRS416-GFP-Dnf1 S611A |  |
| pRS416-GFP-Dnf1 YQS-FSN | (Baldridge and Graham, 2012) |
| pRS416-GFP-Dnf1 N1226A |  |
| pRS425 Lem3 | (Baldridge and Graham, 2012) |
| pRS425 Lem3 (2-49)∆ |  |
| pRS425 Lem3 (400-414)∆ |  |
| pRS425 Lem3 R51A |  |
| pRS313-FLAG-Dnf1 | (Baldridge and Graham, 2012) |
| pRS313-Dnf1 R264A |  |
| pRS313-Dnf1 Q610A |  |
| pRS313-Dnf1 S611A |  |
| pRS313-Dnf1 W652S |  |
| pRS313-Dnf1 N1226A |  |
