# Supplementary material for: Transport mechanism of P4 ATPase phosphatidylcholine flippases
Source: eLife. 2020 Dec 15;9:e62163. doi: 10.7554/eLife.62163 (PMC7773333; doi:10.7554/eLife.62163)
Supplement: Supplementary file 3. [file elife-62163-supp3.docx]

**Supplementary File 3. List of strains used in the study (knockouts are *KanMX* replacements unless otherwise indicated).**

| **Strain** | **Genotype** | **Plasmid** | **Source** |
| --- | --- | --- | --- |
| BY4741 | *MAT****a*** *his3Δ1 leu2Δ0 ura3Δ0 met15Δ0* |  | Invitrogen &  Horizon Discovery |
| PFY3275F | MAT**a** *his3*Δ*1 leu2*Δ*0 ura3*Δ*0* *met15*Δ*0 dnf1*Δ *dnf2*Δ |  | (Hua and Graham, 2003) |
| RBY9701 | PFY3275F *lem3∆*::natNT2 |  | (Baldridge and Graham, 2012) |
| ZHY704 | *MATα his3∆1 leu2∆0 ura3∆0 lys2∆0 dnf1∆ dnf2∆ dnf3∆ drs2∆::LEU2* pRS416-*DRS2* | pRS416-*DRS2* | (Hua et al., 2002) |
| SCY119 | *MATα his3Δ1 leu2Δ0 ura3Δ0 met15Δ0 lem3∆::KanMX* |  | (Baldridge and Graham, 2012) |
| BJ201 | PFY3275F | pRS313 |  |
| BJ204 | PFY3275F | pRS313-Dnf1 |  |
| BJ209 | PFY3275F | pRS313-Dnf1R264A |  |
| BJ212 | PFY3275F | pRS313-Dnf1Y633A |  |
| BJ215 | PFY3275F | pRS313-Dnf1Y633G |  |
| BJ218 | PFY3275F | pRS313-Dnf1T648A |  |
| BJ219 | PFY3275F | pRS313-Dnf1T648V |  |
| BJ222 | PFY3275F | pRS313-Dnf1W652A |  |
| BJ225 | PFY3275F | pRS313-Dnf1W652S |  |
| BJ230 | PFY3275F | pRS313-Dnf1Q610A |  |
| BJ231 | PFY3275F | pRS313-Dnf1S611A |  |
| BJ234 | PFY3275F | pRS313-Dnf1YQS-FSN | (Baldridge and Graham, 2012) |
| BJ237 | PFY3275F | pRS313-Dnf1N1226A |  |
| BJ240 | PFY3275F | pRS313-Dnf1(692-737)∆ |  |
| BJ241 | PFY3275F | pRS313-Dnf1(692-737)∆ |  |
| BJ242 | PFY3275F | pRS313-Dnf1(692-737)∆ |  |
| BJ245 | PFY3275F | pRS416-GFP-Dnf1 |  |
| BJ248 | PFY3275F | pRS416-GFP-Dnf1 R264A |  |
| BJ251 | PFY3275F | pRS416-GFP-Dnf1 W652A |  |
| BJ254 | PFY3275F | pRS416-GFP-Dnf1 W652S |  |
| BJ255 | PFY3275F | pRS416-GFP-Dnf1 Q610A |  |
| BJ260 | PFY3275F | pRS416-GFP-Dnf1 S611A |  |
| BJ263 | PFY3275F | pRS416-GFP-Dnf1 YQS-FSN | (Baldridge and Graham, 2012) |
| BJ266 | PFY3275F | pRS416-GFP-Dnf1 N1226A |  |
| BJ269 | ZHY704 | pRS313 |  |
| BJ272 | ZHY704 | pRS313-Dnf1 |  |
| BJ275 | ZHY704 | pRS313-Dnf1R264A |  |
| BJ276 | ZHY704 | pRS313-Dnf1Y633A |  |
| BJ279 | ZHY704 | pRS313-Dnf1Y633G |  |
| BJ282 | ZHY704 | pRS313-Dnf1T648A |  |
| BJ285 | ZHY704 | pRS313-Dnf1T648V |  |
| BJ288 | ZHY704 | pRS313-Dnf1W652A |  |
| BJ291 | ZHY704 | pRS313-Dnf1W652S |  |
| BJ294 | ZHY704 | pRS313-Dnf1Q610A |  |
| BJ297 | ZHY704 | pRS313-Dnf1S611A |  |
| BJ300 | ZHY704 | pRS313-Dnf1YQS-FSN | (Baldridge and Graham, 2012) |
| BJ303 | ZHY704 | pRS313-Dnf1N1226A |  |
| BJ308 | ZHY704 | pRS313-Dnf1(692-737)∆ |  |
| BJ309 | RBY9701 | pRS313, pRS425 |  |
| BJ310 | RBY9701 | pRS313-Dnf1, pRS425-Lem3 |  |
| BJ311 | RBY9701 | pRS313-Dnf1, pRS425-Lem3 (2-49)∆ |  |
| BJ312 | RBY9701 | pRS313-Dnf1, pRS425-Lem3 (400-414)∆ |  |
| BJ313 | RBY9701 | pRS313-Dnf1, pRS425-Lem3 R51A |  |
| BJ314 | SCY119 | pRS416-GFP-Dnf1, pRS425 |  |
| BJ315 | SCY119 | pRS416-GFP-Dnf1, pRS425-Lem3 |  |
| BJ316 | SCY119 | pRS416-GFP-Dnf1, pRS425-Lem3 (2-49)∆ |  |
| BJ317 | SCY119 | pRS416-GFP-Dnf1, pRS425-Lem3 (400-414)∆ |  |
| BJ318 | SCY119 | pRS416-GFP-Dnf1, pRS425-Lem3 R51A |  |
| BJ319 | SCY119 | pRS313-FLAG-Dnf1, pRS425 |  |
| BJ322 | SCY119 | pRS313-FLAG-Dnf1, pRS425-Lem3 |  |
| BJ325 | SCY119 | pRS313-FLAG-Dnf1, pRS425-Lem3 (2-49)∆ |  |
| BJ328 | SCY119 | pRS313-FLAG-Dnf1, pRS425-Lem3 (400-414)∆ |  |
| BJ331 | SCY119 | pRS313-FLAG-Dnf1, pRS425-Lem3 R51A |  |
| BJ334 | PFY3275F | pRS313-FLAG-Dnf1 | (Baldridge and Graham, 2012) |
| BJ337 | PFY3275F | pRS313-FLAG-Dnf1 R264A |  |
| BJ340 | PFY3275F | pRS313-FLAG-Dnf1 Q610A |  |
| BJ343 | PFY3275F | pRS313-FLAG-Dnf1 S611A |  |
| BJ346 | PFY3275F | pRS313-FLAG-Dnf1 W652S |  |
| BJ349 | PFY3275F | pRS313-FLAG-Dnf1 N1226A |  |
