# Supplementary material for: Transport mechanism of P4 ATPase phosphatidylcholine flippases
Source: eLife. 2020 Dec 15;9:e62163. doi: 10.7554/eLife.62163 (PMC7773333; doi:10.7554/eLife.62163)
Supplement: Transparent reporting form [file elife-62163-transrepform.docx]

***eLife’s* transparent reporting form**

We encourage authors to provide detailed information *within their submission* to facilitate the interpretation and replication of experiments. Authors can upload supporting documentation to indicate the use of appropriate reporting guidelines for health-related research (see [EQUATOR Network](http://www.equator-network.org/%20)), life science research (see the [BioSharing Information Resource](https://biosharing.org/)), or the [ARRIVE guidelines](http://www.plosbiology.org/article/info:doi/10.1371/journal.pbio.1000412) for reporting work involving animal research. Where applicable, authors should refer to any relevant reporting standards documents in this form.

If you have any questions, please consult our Journal Policies and/or contact us: [editorial@elifesciences.org](mailto:editorial@elifesciences.org).

**Sample-size estimation**

- You should state whether an appropriate sample size was computed when the study was being designed
- You should state the statistical method of sample size computation and any required assumptions
- If no explicit power analysis was used, you should describe how you decided what sample (replicate) size (number) to use

Please outline where this information can be found within the submission (e.g., sections or figure legends), or explain why this information doesn’t apply to your submission:

There are cryo-EM structures in this report. The appropriate sample sizes were computed, as shown and described in both the methods section and the appropriate figure legends.

**Replicates**

- You should report how often each experiment was performed
- You should include a definition of biological versus technical replication
- The data obtained should be provided and sufficient information should be provided to indicate the number of independent biological and/or technical replicates
- If you encountered any outliers, you should describe how these were handled
- Criteria for exclusion/inclusion of data should be clearly stated
- High-throughput sequence data should be uploaded before submission, with a private link for reviewers provided (these are available from both GEO and ArrayExpress)

Please outline where this information can be found within the submission (e.g., sections or figure legends), or explain why this information doesn’t apply to your submission:

Sample sizes for the cryo-EM are large data sets, and the process by which they were worked up are shown in Methods. Biochemical experiments contained either 3 trials for each datapoint as shown and described in the methods and legends.

**Statistical reporting**

- Statistical analysis methods should be described and justified
- Raw data should be presented in figures whenever informative to do so (typically when N per group is less than 10)
- For each experiment, you should identify the statistical tests used, exact values of N, definitions of center, methods of multiple test correction, and dispersion and precision measures (e.g., mean, median, SD, SEM, confidence intervals; and, for the major substantive results, a measure of effect size (e.g., Pearson's r, Cohen's d)
- Report exact p-values wherever possible alongside the summary statistics and 95% confidence intervals. These should be reported for all key questions and not only when the p-value is less than 0.05.

Please outline where this information can be found within the submission (e.g., sections or figure legends), or explain why this information doesn’t apply to your submission:

Analysis of cryo-EM 3D map resolutions and p values, where appropriate, is described in the methods legends and figures.

(For large datasets, or papers with a very large number of statistical tests, you may upload a single table file with tests, Ns, etc., with reference to sections in the manuscript.)

**Group allocation**

- Indicate how samples were allocated into experimental groups (in the case of clinical studies, please specify allocation to treatment method); if randomization was used, please also state if restricted randomization was applied
- Indicate if masking was used during group allocation, data collection and/or data analysis

Please outline where this information can be found within the submission (e.g., sections or figure legends), or explain why this information doesn’t apply to your submission:

Complete description of how particles were sorted and selected, alone with the FSC curves coverage of particle orientation over the samples are given in the methods and supplemental figures.

**Additional data files (“source data”)**

- We encourage you to upload relevant additional data files, such as numerical data that are represented as a graph in a figure, or as a summary table
- Where provided, these should be in the most useful format, and they can be uploaded as “Source data” files linked to a main figure or table
- Include model definition files including the full list of parameters used
- Include code used for data analysis (e.g., R, MatLab)
- Avoid stating that data files are “available upon request”

Please indicate the figures or tables for which source data files have been provided:

Accession codes. The cryo-EM 3D maps and the corresponding atomic models of the Dnf1–Lem3 complex have been deposited at the EMDB database and the RCSB PDB with the respective accession codes of EMD-23069 and 7KY6 (apo E1), EMD-23074 and 7KYB (E1P-ADP), EMD-23077 and 7KYC (E2P). The cryo-EM 3D maps and the corresponding atomic models of the Dnf2–Lem3 complex have been deposited at the EMDB database and the RCSB PDB with the respective accession codes of EMD-23070 and 7KY7 (apo E1), EMD-23071 and 7KY8 (E1-ATP), EMD-23072 and 7KY9 (E1P-ADP), EMD-23068 and 7KY5 (E2P-transition), and EMD-23073 and 7KYA (E2P).
